# Outlier Profiles of Atomic Structures Derived from X-ray Crystallography and from Cryo-Electron Microscopy

**DOI:** 10.3390/molecules25071540

**Published:** 2020-03-28

**Authors:** Lin Chen, Jing He

**Affiliations:** 1 Department of Computer Science, Valdosta State University, 1500 N Patterson St, Valdosta, GA 31698, USA; 2 Department of Computer Science, Old Dominion University, 5115 Hampton Blvd, Norfolk, VA 23529, USA; jhe@cs.odu.edu

**Keywords:** protein structure, cryo-electron microscopy, validation, statistics, X-ray, machine learning, sidechain, anomaly

## Abstract

*Background:* As more protein atomic structures are determined from cryo-electron microscopy (cryo-EM) density maps, validation of such structures is an important task. *Methods:* We applied a histogram-based outlier score (HBOS) to six sets of cryo-EM atomic structures and five sets of X-ray atomic structures, including one derived from X-ray data with better than 1.5 Å resolution. Cryo-EM data sets contain structures released by December 2016 and those released between 2017 and 2019, derived from resolution ranges 0–4 Å and 4–6 Å respectively. *Results:* The distribution of HBOS values in five sets of X-ray structures show that HBOS is sensitive distinguishing sets of X-ray structures derived from different resolution ranges-higher than 1.5 Å, 1.5–2.0 Å, 2.0–2.5 Å, 2.5–3.0 Å, and 3.0–3.5 Å. The overall quality of cryo-EM structures is likely improved, as shown in a comparison of cryo-EM structures released before the end of 2016, those between 2017 and 2018, and those between 2018 and 2019. Our investigation shows that leucine (LEU) has a significantly higher rate of HBOS outliers than that of the reference data set (X-ray-1.5) and of other residue types in the cryo-EM data sets. HBOS was able to detect outliers for those residues that are currently marked as green in PDB validation reports. *Conclusions:* The HBOS profile of a dataset is a potential method to characterize the overall structural quality of the set. Residue LEU deserves special attention since it has a significantly higher HBOS outlier rate in sets of cryo-EM structures and those X-ray structures derived from X-ray data of lower than 2.5 Å resolutions. Most HBOS outlier residues from the EM-0-4-2019 set are located on loops for most types of residues.

## 1. Introduction

Cryo-electron microscopy (cryo-EM) is an essential method to determine three-dimensional atomic structures of proteins and some RNA and DNA molecules [1,2,3,4,5,6,7]. Unlike X-ray crystallography, cryo-EM technique is not limited by crystallization of proteins, and hence it suits a broader range of molecules [8]. Molecules are quickly frozen in solution and are imaged in near-native conditions [9]. As of January 08, 2020, the Worldwide Protein Data Bank (wwPDB) (https://www.wwpdb.org/) contains 4189 entries of atomic structures derived from the cryo-EM technique, about 2.6% of 159,230 structures in wwPDB [10,11]. Developing validation strategies for cryo-EM models has become one of the major challenges in the cryo-EM community. 

To develop standards, formats, and specifications for proteins, the Protein Data Bank community convened three Validation Task Force (VTF) for X-ray [12], NMR [13], and EM [14] from 2010 to 2012. The EM VTF report, published in 2012, reflects suggestions from both experimental and modeling communities in the following three components: (1) the assessment of models using constraints (geometry, conformation, and molecular interactions) from known molecular models with and without regard to density maps; (2) validation of EM density maps and creation of standards to assess image quality; (3) development of software to assist validation [14].

Various methods have been developed to validate experimental data, protein models, and the fit between experimental data and protein models [15,16,17,18,19,20]. In the OneDep structure deposition system, a validation report is derived for each structure using X-ray, NMR, or cryo-EM validation criteria [21]. The overall quality of a protein model and individual residues are analyzed using MolProbity and a few other software packages [20,22,23,24]. Validation reports for protein structures are downloadable from wwPDB [21]. The metric of overall quality in an EM validation report contains clashscore, Ramachandran outliers, sidechain outliers, and RNA backbone. The percentile rank is provided for each category after comparing the validated structure with the entire PDB archive and cryo-EM structures, respectively. Outlier residues are detected after considering ideal values of bonds and angles, torsion angle statistics, and contact distances [21]. In the residue-property plot section of the validation report, a residue is color-coded by the number of outlier types. Green, yellow, orange, and red colors are used to represent respectively 0, 1, 2, and 3 or more types of outliers. Note that outliers do not mean errors in the model. Outliers may be genuine, unusual, and of biological interest, but they deserve attention. 

We previously observed that block length is a simple but sensitive measure of sidechain conformations [25]. In a histogram-based outlier score (HBOS) method, we introduced two distance measures, block length and sidechain distance, that are not used in the current OneDep validation system [26,27]. In this paper, we report HBOS profiles of six cryo-EM datasets and five X-ray datasets at different resolution ranges. HBOS uses a different way to combine multiple measurements than used in the OneDep validation method. Two of the five features measured in HBOS are not used OneDep validation system. HBOS profiles represent alternative characterization of residue conformations than what is provided in the OneDep validation report. 

## 2. Results

### 2.1. Eleven Sets of Atomic Structures Derived from X-ray and Cryo-EM Data

This study utilizes a reference set of protein structures that are derived from X-ray data of the highest resolutions since those structures are expected to have the highest accuracy. The reference dataset, X-ray-1.5, was constructed from 9131 protein structures that are derived from X-ray data with resolutions better than 1.5 Å. At the resolution 1.5 Å, major atoms in a protein are well identified. Four other X-ray datasets X-ray-1.5–2.0, X-ray-2.0–2.5, X-ray-2.5–3.0, and X-ray-3.0–3.5 contain structures derived from X-ray data with resolutions between 1.5 Å to 2.0 Å, 2.0 Å to 2.5 Å, 2.5 to 3.0 Å, and 3.0 to 3.5 Å respectively (Table 1). Protein structures in five X-ray datasets were downloaded from RCSB PDB [28] website in March 2018 with a sequence similarity of less than 90%. RCSB PDB is a member of the wwPDB. Since non-crystallographic symmetry is commonly seen in a protein structure, chains with 95% sequence identity with any other chains in the same protein are ignored to avoid duplication. Since there are over 20,000 available structures derived from X-ray data with resolution range of 1.5–2.0Å, 2.0–2.5 Å, and 2.5–3.0 Å respectively, 5000 structures from each of the three resolution ranges were randomly selected to compose the datasets.

Local resolution methods measure resolution at each voxel of a density map and hence provide an estimation of resolution locally [29,30]. However, the resolution of the entire density map is a single number for a rough estimation of the overall quality of the density map. We binned cryo-EM density maps into six groups using the resolution of the maps and their release time (Table 1). As an example, EM-0-4-2016 and EM-0-4-2019 contain 213 and 1175 atomic structures derived from cryo-EM density maps with 0–4 Å resolution that are released before March 31, 2016 and those between April 1, 2018 and December 31, 2019 respectively. Since there is continuing effort at PDB to update deposited structures, the number of obsolete entries that are used in the eleven datasets are indicated in Table 1. We observed that the largest numbers of obsolete entries among the eleven sets are 138 in X-ray-3.0-3.5 and 138 in EM-0-4-2019.

### 2.2. HBOS Distribution of X-ray and Cryo-EM datasets

HBOS is an outlier score that measures the distribution of five geometrical features of a residue in a protein—backbone torsion angle Phi (φ) and Psi (ψ), sidechain torsion angle ($\chi_{1}$), sidechain length ($d_{sidechain}$), and block length ($d_{block}$) (see a summary in 4.2) [26]. An unpopular geometry shown in one or more of the five features is reflected by a high HBOS value that does not necessarily imply a wrong configuration. However, the distribution of HBOS values in a population represents the systematic characteristics of the population, given that there is a sufficient number of residues in the dataset. The difference among HBOS distributions across different populations may suggest the overall quality difference among those populations. 

**Figure 1:**
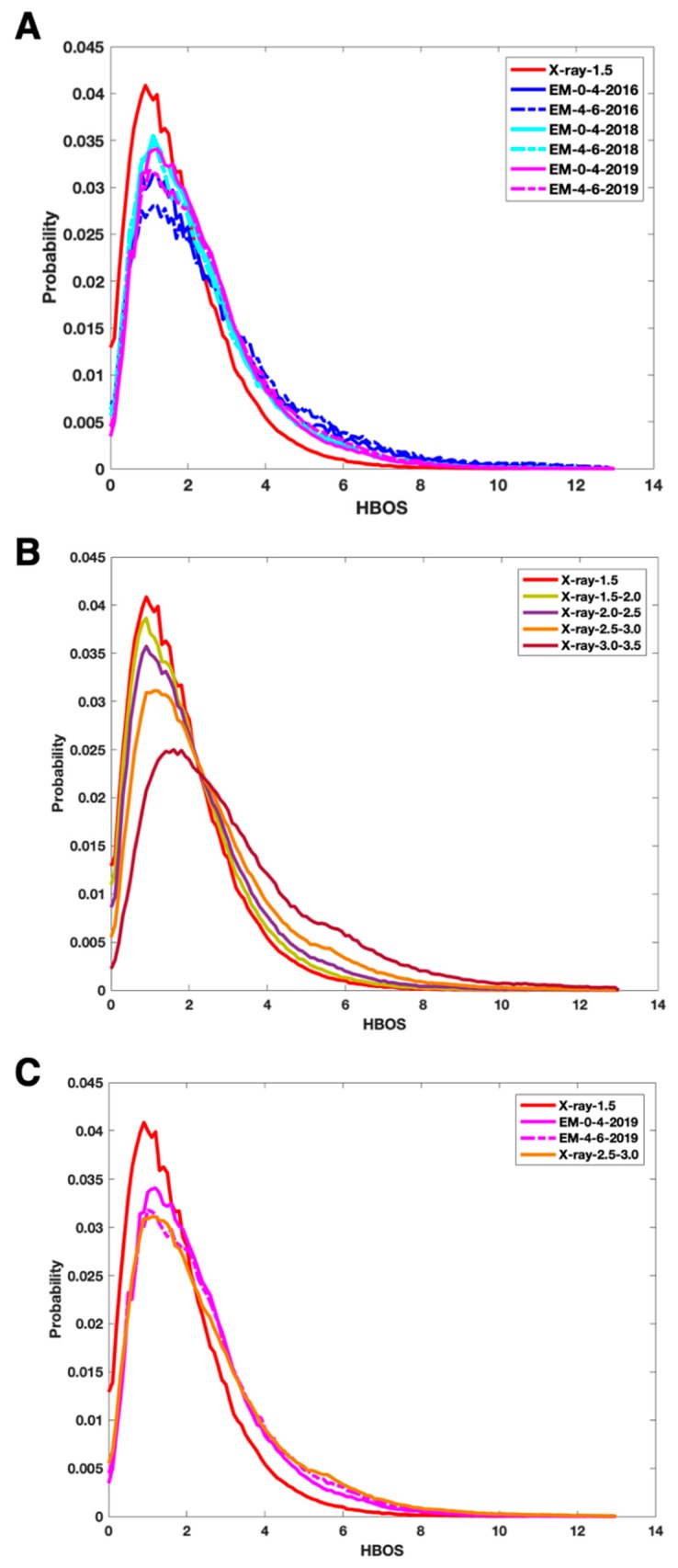
Probability histogram plots for seven datasets. (**A**) The probability histogram of residue HBOS scores in X-ray-1.5 (red solid), EM-0-4-2016 (blue solid), EM-4-6-2016 (blue dash line), EM-0-4-2018 (cyan solid), EM-4-6-2018 (cyan dash line), EM-0-4-2019 (magenta solid), and EM-4-6-2019 (magenta dash line). (**B**) The probability histogram of residue HBOS scores in X-ray-1.5 (red solid), X-ray-1.5–2.0 (olive solid), X-ray-2.0–2.5 (purple solid), X-ray-2.5–3.0 (orange solid), and X-ray-3.0–3.5 (maroon solid). For clear viewing, four curves are shown for X-ray-1.5, EM-0-4-2019, EM-4-6-2019, and X-ray-2.5–3.0 in (**C**).

We investigated the distribution of HBOS for each of the five sets of X-ray structures (Figure 1B). A probability histogram was derived by normalizing the distribution of HBOS by the area under the curve (Figure 1). The bin size of the histogram plot is 0.1. Since the five probability histogram curves have the same area, 1, under each curve, the height of a peak represents the popularity of the HBOS value at the peak, independent from the size of the dataset. We observed that the most popular HBOS value (at the peak) among the five X-ray sets are between 0 and 2. The height of the peaks strictly reduces as the resolution of the dataset increases, with X-ray-1.5 set having the highest peak and X-ray-3.0–3.5 having the lowest peak. This suggests that it is more popular to have a smaller HBOS value (between 0 and 2) in the X-ray-1.5 set than for other datasets. Normalized HBOS distribution can distinguish the effect of the resolution of X-ray data from which atomic structures are derived. Generally, the higher resolution of X-ray data is expected to produce a higher quality of structures. The reference dataset X-ray-1.5 has the skinniest curve indicating that it is extremely rare to have a residue with a large HBOS value. In fact, only 0.088% of the residues in the entire dataset has HBOS scores greater than 10 (Figure 1B). However, 0.737% of EM-0-4-2016 and 1.44% of EM-4-6-2016 set have HBOS values greater than 10.

Since X-ray-1.5 dataset contains protein structures derived from the highest resolutions, we used its probability histogram as a reference. The probability histogram for X-ray-1.5 has a peak at the HBOS value of 0.9 with a probability of 0.04 (red solid curve in Figure 1). The two curves with the lowest peaks are EM-4-6-2016 (blue dash) and EM-0-4-2016 (blue solid) with the probability of 0.028 and 0.03 respectively for the peak (near HBOS score of 1.1) (Figure 1A). The lower probability for the HBOS value at the peak suggests a higher probability for larger HBOS values, as shown at the tail of the curves. We observe that EM-0-4-2018 (cyan solid) and EM-0-4-2019 (magenta solid) show curves closer to the curve of X-ray-1.5 than that of EM-0-4-2016 (Figure 1A). It suggests that the quality of cryo-EM protein structures have improved since 2017. More rigorous structure determination standards for cryo-EM structures might have contributed to the improvement. In fact, the probability histogram curve of EM-0-4-2019 is the closest to that of X-ray-2.5–3.0 (Figure 1C) among the five curves of X-ray data (Figure 1B). The height of the peak in EM-0-4-2019 curve is slightly higher than that of X-ray-2.5–3.0, but it is still lower than that of X-ray-2.0–2.5. It suggests that the overall structural quality of the EM-0-4-2019 set is at least comparable to that of X-ray-2.5–3.0. Given that most of the cryo-EM density maps for the EM-0-4-2019 set have resolutions between 3 Å and 4 Å, it is impressive to see the current structure determination method produces overall quality comparable to that of X-ray structures derived from data of 2.5–3.0 Å resolutions. The HBOS curve (magenta solid) of EM-0-4-2019 is almost identical to the curve of EM-0-4-2018 (cyan solid). The similarity of the two curves suggests similar standards/software being used in structure determination from 2017 to 2019. 

It is noted that almost identical curves were observed for EM-0-4-2018 and EM-4-6-2018. This suggests that the resolution difference, 0–4 Å versus 4–6 Å resolutions, does not make much difference producing structure outliers among the two datasets. Since most structures derived from density maps with 4–6 Å resolutions use known structures as the template, it is not clear how much the refinement depends on the density maps. The curve (magenta dash) for EM-4-6-2019 has the value 0.031 at peak position HBOS 1.2, which is lower than the peak value 0.034 for EM-0-4-2019. This is expected if the refinement of a template structure utilizes a density map that may or may not provide enough details about side chains at 4–6 Å resolutions.

### 2.3. Histogram-based Outliers of Different Residue Types

**Figure 2:**
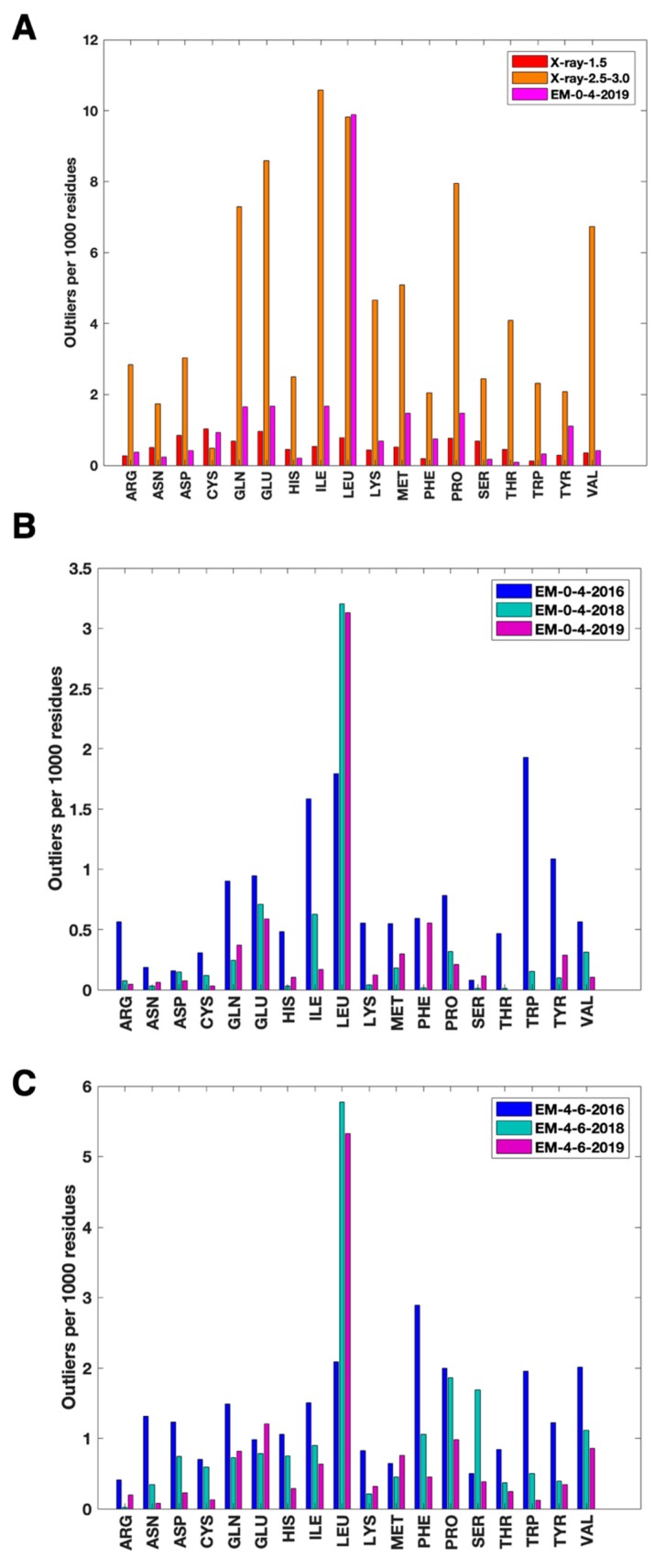
HBOS outliers of various residue types. (**A**) The number of outlier residues with HBOS greater than 10 per 1000 same type of residues in X-ray-1.5 (red), X-ray-2.5–3.0 (orange), and EM-0-4-2019 (magenta). (**B**) The number of HBOS outlier residues (with HBOS greater than 10) that are labeled green in wwPDB validation reports per 1000 same type of residues in EM-0-4-2016 (blue), EM-0-4-2018 (cyan), and EM-0-4-2019 (magenta); (**C**) Similar information as in (**B**) for three different datasets: EM-4-6-2016 (blue), EM-4-6-2018 (cyan), EM-4-6-2019 (magenta).

To understand the nature of those residues with high HBOS values, we investigated 18 of 20 types of residues with HBOS values larger than 10. Two types of residues, glycine (GLY) and alanine (ALA), are ignored since they have no $\chi_{1}$ due to their small sizes of sidechains. We observed that it is extremely rare for a residue to have an HBOS value larger than 10 in the reference dataset. In this study, an HBOS outlier refers to a residue with an HBOS value greater than 10, an empirical value for investigation of such cases. For the reference dataset, X-ray-1.5, all of the 18 residue types have lower than 1 outlier per 1000 residues of the same type (red in Figure 2A). For EM-0-4-2019, the dataset with most-recently determined cryo-EM structures, leucine (LEU) has about 9.89 occurrence rate, significantly higher than that of the reference set (Figure 2A). In a scan to the X-ray-2.5–3.0 set, LEU also shows a similar occurrence rate as in EM-0-4-2019. The high outlier occurrence rate of LEU may indicate a problem in the structure determination of an LEU residue for the density maps involved. Five other residue types in the EM-0-4-2019 set have nearly twice outlier rates as that of the reference set: glutamic acid (GLU), glutamine (GLN), isoleucine (ILE), methionine (MET), proline (PRO), and tyrosine (TYR). All residue types except for cysteine (CYS) show a significantly higher frequency of outliers in the X-ray-2.5–3.0 set when compared to the reference set (Figure 2A). 

Each protein structure in PDB has a validation report produced from the OneDep system [21]. OneDep validation system measures various features, but HBOS only measures five. We expect that OneDep to be a general validation system that identifies a broad spectrum of outliers. However, HBOS has a unique way to measure residue configurations, and it is an independent metric that may be sensitive in detecting outliers in certain situations. We investigated those residues that are labeled green in PDB validation reports but have large HBOS values. Note that a residue is labeled green in the validation reports if there are zero outlier types identified in the OneDep system. The difference in the outlier occurrence rate of 9.89 (Figure 2A) and 3.13 (Figure 2B) for LEU shows that most of HBOS outliers for LEU are indicated in PDB validation reports, shown as non-green colors. 

However, 3.13 per 1000 leucine residues are still considered as HBOS outliers, and they marked green in PDB validation reports. This occurrence rate is still significantly higher than the rate of LEU in the reference set and is also the highest rate among all residue types investigated. Results show that HBOS is potentially more sensitive in detecting certain characters than what is currently implemented in the wwPDB validation system. Further investigation is needed to understand the nature of high-risk configurations of residues and to explore the potential of using HBOS as a complementary measure for the normal residues indicated in PDB validation reports. 

For those residues that are marked green in PDB validation reports, we observed a significant decrease in the HBOS outlier rate from EM-0-4-2016 to EM-0-4-2019 for tryptophan (TRP), TYR, PRO, ILE, lysine (LYS), histidine (HIS), GLN, and arginine (ARG) (Figure 2B). This suggests that HBOS finds less outliers in the 2019 set than in the 2016 set among those residues that are considered normal in PDB validation reports. In fact, almost all residue types have reduced outlier rates except LEU, phenylalanine (PHE), and serine (SER), although the rate for SER is already low (Figure 2B). As an example, the height of the ARG bar is 0.56 in the EM-0-4-2016 set, which is much higher than 0.08 in the EM-0-4-2018 set and 0 in the EM-0-4-2019 set. The sidechain quality of ARG might have been improved over time. A similar trend in the reduction of HBOS outlier rate among normal residues in PDB validation reports was also observed for the two 4-6 Å resolution sets (Figure 2C). This may suggest the improved quality in sidechain conformations. Most of the residue types in the EM-4-6-2019 set (purple in Figure 2C) have higher HBOS outlier rates than the corresponding residue types in the EM-0-4-2019 set (purple in Figure 2B). This aligns with the observation that it is in general harder to determine structure precisely from a 4–6 Å resolution density map than from a 0–4 Å resolution map. 

**Figure 3:**
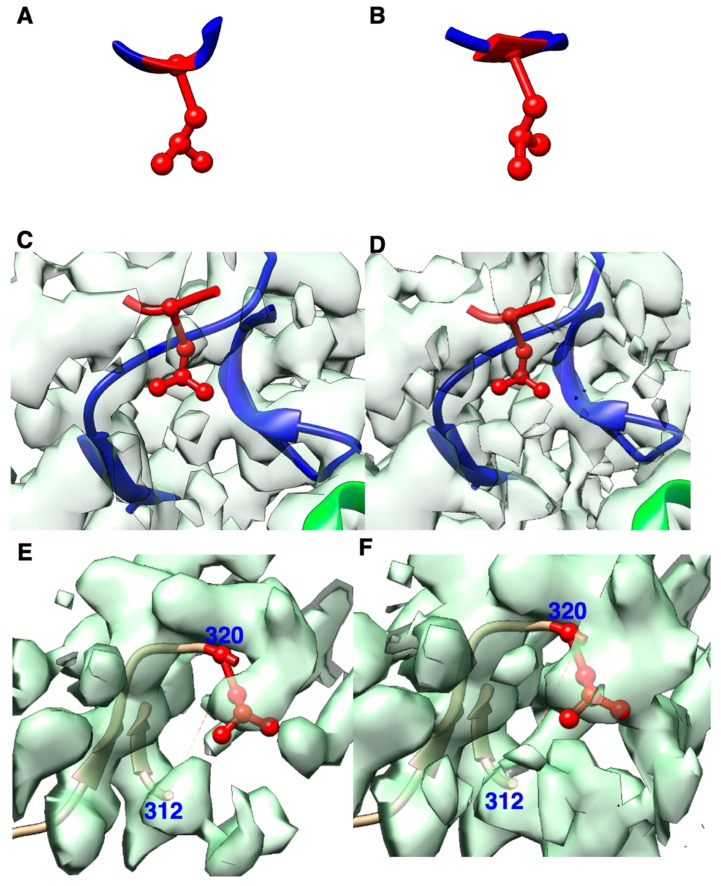
Four leucine (LEU) examples. (**A**) LEU with index 17 in chain A of protein 6g72 (PDB ID), not an HBOS outlier; (**B**) LEU with index 167 in chain L of protein 6j0n (PDB ID), an HBOS outlier with abnormally long sidechain length; (**C**,**D**) LEU with index 409 in chain X of protein 6j0n (PDB ID) superimposed on cryo-EM density maps using density threshold 0.1 in (**C**) and 0.07 in (**D**), an HBOS outlier having abnormally short sidechain length; (**E**,**F**) LEU with index 320 in chain A of protein 6fe8 (PDB ID) is superimposed on the cryo-EM density map using a threshold of 0.06 in (**E**) and 0.04 in (**F**), an HBOS outlier having abnormal backbone torsion angle. The segment from 313 to 319 is not available in the structure.

In order to visualize conformations of LEU outliers, we sampled four LEU configurations that are all marked green in PDB validation reports. One of the four (Figure 3A) is not an HBOS outlier, and the other three are (Figure 3B–F). The normal configuration in Figure 3A has $d_{sidechain}$ and $d_{block}$ as 2.58 Å and 3.1 Å respectively, both near popular distances for $d_{sidechain}$ and $d_{block}$. Two features, $d_{sidechain}$ and $d_{block}$, of the five features measured are abnormal for the LEU in Figure 3B. Its $d_{sidechain}$ and $d_{block}$ are 2.79 Å and 3.25 Å respectively, unusually long. In this case (Figure 3B), CD1 atom and CD2 atom are father from the backbone than normal, even though φ, ψ, and χ angle values are still in the acceptable bin. The probability of observing such a conformation is near zero in the reference set (X-ray-1.5). In another HBOS outlier (Figure 3C,D), the triangle of CG-CD1-CD2 is bent towards the backbone with unusually short values for $d_{sidechain}$ (2.38 Å) and $d_{block}$ (2.78 Å). As shown in Figure 3E, the structure of a segment of seven residues is not included in the model between residue 312 to 320. The missing segment in the model provides extra space and potentially unclaimed density to allocate the sidechain of LEU 320. The sidechain of LEU 320 appears to fit well in the density cloud nearby and presumably has a good fitting score. The conformation of LEU 320 has reasonable $d_{sidechain}$, $d_{block}$, and χ values, but it has an unfavorite ψ value on the backbone. 

### 2.4. HBOS Outliers on Secondary Structures

The secondary structures of HBOS outlier residues in the EM-0-4-2019 dataset were analyzed according to the classification of Dictionary of Protein Secondary Structure (DSSP) [31]. A similar analysis for X-ray-1.5 and the other five EM datasets are provided in Supplementary Materials (Tables S1–S6). The coil, bend, and turn together, if referred to as loops, contain 64.66% of HBOS outliers in the EM-0-4-2019 set, much higher than that of 24.36% for helices (Table 2). 14 of 18 residue types have over 50% of HBOS outliers on loops, except for HIS, PHE, SER, and TYR. Residues on loops may have more flexibility to adopt a sidechain conformation, and it is perhaps also harder to determine conformations under limited constraints. For HIS and TYR, the most outliers are on β-sheets (E and B labels included). Their large sidechains might be the challenge to be assigned favorite conformations on β-sheets. Most of the outliers of PHE and SER are on Helix. 

A comparison between Table 2 (EM-0-4-2019) with Table S1 (X-ray-1.5) shows that the cryo-EM structures have a 10% higher percentage of outliers on coils than that of the X-ray structures for 10 types of residues - ASN, ASP, GLN, GLU, LYS, MET, THR, TRP, TYR, and VAL. Most of those residue types have large sidechains except for VAL. For example, 60.98% of ASP outliers are on coils in EM-0-4-2019, but only 24.34% ASP outliers are on coil in X-ray-1.5 (Table S1). In contrast, EM-0-4-2019 has a lower percentage of outliers on turns than X-ray-1.5 for 15 of the 18 residue types, such as ASN, ASP, CYS, GLN, HIS, ILE. 

## 3. Materials and Methods 

### 3.1. Datasets

The Python scripts used in this study have been deposited to the Github repository at https://github.com/lin-chen-VA/MDPI_Molecules_2020. The Python source code and tutorials of analysis tools have also been added with their flowcharts to the repository. The protein structures in the six cryo-EM datasets were downloaded in cif format from RCSB PDB. As a wwPDB archive keeper, RCSB PDB synchronizes the PDB archive at ftp://ftp.wwpdb.org. Since the function of searching cryo-EM proteins by resolution has been removed from wwPDB in early 2018, the proteins in each dataset were downloaded with a web downloader provided by RCSB PDB (https://www.rcsb.org/pdb/download/download.do) followed by a resolution filter module (resolution.py in the script package) for a specific resolution range. Since wwPDB is continuously updated when a new structure of the same protein chain is available, some proteins in the datasets are obsolete after datasets were created. 

### 3.2. Histogram-Based Outlier (HBOS)

**Figure 4:**
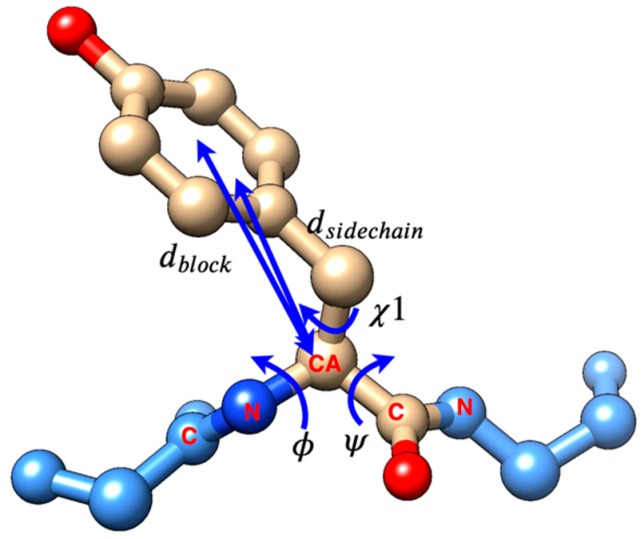
The five features φ, ψ, $\chi_{1}$, $d_{\mathrm{sidechain}}$ and $d_{\mathrm{block}}$ of a protein residue.

The torsional angle φ is formed by atom C in the previous residue in the protein sequence chain and atom N, atom CA, atom C in the current residue (Figure 4). The ψ is formed by atom N, atom CA, atom C in the current residue and atom N in the next residue in the sequence. The $\chi_{1}$ angle is the first torsion angle in the sidechain, which is formed by atoms N, CA, CB, and CG. The range of three torsion angles we implemented in the code is 0°–360°, instead of −180°–+180° in the Ramachandran plot [32]. $d_{sidechain}$ is the distance between CA atom on the backbone and the mass centroid of the sidechain. $d_{block}$ is the distance between the CA atom on backbone and mass centroid of the distal block of a specific residue. The blocks in a residue are defined in Chen et al. [25]. Histogram-based outlier score (HBOS) of each residue was calculated by equation (1). HBOS is an unsupervised model based on the idea of Naïve Bayes (see more details in Chen et al. [27]).

(1) $$HBOS_{j}\left(v_{1},v_{2},v_{3,}v_{4},v_{5}\right)=\sum_{i=1}^{5}HBOS_{j}\left(v_{i}\right)=\sum_{i=1}^{5}log\left(\frac{1}{npdf_{i,\;j}\left(v_{i}\right)}\right)$$

Let $npdf_{i,\;j}$ ($v_{i}$) be the normalized density function value for feature *i* and residue *j* when $i=v_{i}$. For example, $npdf_{d_{Block,Lys}}$ (3.0) is the function value when $d_{Block}$ = 3.0 Å for LYS. The HBOS score of a residue is the summation of the five HBOS values from the five features. A residue with a high HBOS has a low probability of occurrence, and its conformation is unfavorable. A residue with an HBOS score greater than 10 is considered as an outlier in this paper. The detection of outlier residues was conducted with an outlier detection module (detection.py in the script package).

### 3.3. Outliers in PDB Validation Reports

For each protein structure, its validation report is accessible at RCSB PDB. Besides the pdf version of the validation report, RCSB PDB maintains metadata in XML format. The outliers of different criteria are listed in XML metadata files, such as Ramachandran, rotamer, omega, clashes, bond length, and bond angle [21]. The “OUTLIER” is marked when the OneDep system observes unpopular metrics. For each HBOS outlier residue (labeled by detection.py in the script package), we downloaded the corresponding XML file from ftp://ftp.rcsb.org/pub/pdb/validation_reports/in May 2018 using a web crawler module (labelling.py in the script package), then extracted the outlier information. For cryo-EM models released after May 2018 in EM-0-4-2019 and EM-4-6-2019, their XML validation reports were accessed in January 2020. In validation reports, a residue is color-coded as green if there is no outlier observed in the OneDep system, yellow if there are outliers for one criterion, orange for two criteria, red for three or more criteria. For example, a residue with the mark of rotamer outliers and two atomic clashes is colored in orange. 

### 3.4. Identification of Outlier Secondary Structures

The secondary structures of HBOS outlier residues, reported in Table 2, were identified by a secondary labeling module (DSSP.py in the script package) [31,33]. For each HBOS outlier residue labeled by the detection module (detection.py), the protein structure in PDB format was downloaded for secondary structure analysis, since DSSP in the script package does not support cif format. The identification of secondary structures of residues was conducted in January 2020. The obsolete proteins in the datasets were not used.

## 4. Conclusions

The sidechain centroid and the centroid of the distant block of a residue have been used as the sensitive representation of sidechain conformations in energy functions. We developed a histogram-based metric, HBOS, to characterize the popularity of sidechain conformations. Results from the distribution of HBOS values in five sets of X-ray structures and six sets of cryo-EM structures show that HBOS is sensitive distinguishing sets of X-ray structures derived from different resolution ranges −< 1.5 Å, 1.5–2.0 Å, 2.0–2.5 Å, 2.5–3.0 Å, and 3.0–3.5 Å. Our investigation suggests that the quality of cryo-EM structures has improved when comparing those released before the end of 2016 and those between 2017 and 2018, and those between 2018 and 2019. The probability histogram of the EM-0-4-2019 set is most similar to that of X-ray-2.5–3.0 among the five X-ray sets, suggesting an overall similar level of quality. Our investigation shows that LEU has a significantly higher rate of HBOS outliers than that of the reference dataset (X-ray-1.5) and of other residue types in the cryo-EM datasets. Further investigation is needed to understand the structure determination process for LEU. Since HBOS only targets five features of a residue, it may not be suitable for a general validation method to screen for a variety of outliers. However, we have shown the potential of using it as a complementary screen after PDB validation reports are produced. HBOS was able to detect outliers for those residues that are currently marked as green in validation reports. Such outliers show a higher occurrence rate for cryo-EM structures derived from 4–6 Å resolution than for 0–4 Å resolution density maps. This aligns with the general expectation that it is harder to determine structure precisely from a density map with a 4–6 Å resolution than with a 0–4 Å resolution. Further study is needed to understand the nature of HBOS outliers. 

## Figures and Tables

**Table 1:** Five X-ray atomic structures and six cryo-EM atomic structures used for HBOS profiles. The resolution ranges of X-ray data or cryo-EM density maps are included in the names of the datasets. The number of proteins in the dataset, the number of obsolete proteins as of 3/8/2020, and the release time of the structures are indicated in separate columns.

Dataset	Resolution	Number of Proteins	Number of Obsolete Entries	Release Time
X-ray-1.5	<1.5 Å	9131	2	before 3/31/2018
X-ray-1.5–2.0	1.5–2.0 Å	5000	0	before 3/31/2018
X-ray-2.0–2.5	2.0–2.5 Å	5000	2	before 3/31/2018
X-ray-2.5–3.0	2.5–3.0 Å	5000	22	before 3/31/2018
X-ray-3.0–3.5	3.0–3.5 Å	6833	138	before 3/31/2018
EM-0-4-2016	<4.0 Å	213	47	before 12/31/2016
EM-4-6-2016	4–6 Å	161	19	before 12/31/2016
EM-0-4-2018	<4.0 Å	286	59	1/1/2017 to 3/31/2018
EM-4-6-2018	4–6 Å	158	11	1/1/2017 to 3/31/2018
EM-0-4-2019	<4.0 Å	1175	138	4/1/2018 to 12/31/2019
EM-4-6-2019	4–6 Å	498	52	4/1/2018 to 12/31/2019

**Table 2:** The number of HBOS outliers and the percentage of the EM-0-4-2019 dataset in different secondary structure categories. “-“ (coil), “T” (hydrogen-bonded turn), “S” (bend between two secondary structures), “G” (3-turn helix), “H” (4-turn helix), “I” (5-turn helix), “E”(extended strand in sheets), and “B” (isolated beta-bridge in β-sheets).

	Loop/Turn			Sheet		Helix			-	T	S	Helix	Sheet
	-	S	T	B	E	G	H	I					
ARG	35	13	5	0	0	23	1	0	45.45%	6.49%	16.88%	31.17%	0.00%
ASN	29	16	0	0	9	3	0	0	50.88%	0.00%	28.07%	5.26%	15.79%
ASP	50	11	7	0	5	5	4	0	60.98%	8.54%	13.41%	10.98%	6.10%
CYS	27	12	1	2	6	0	14	0	43.55%	1.61%	19.35%	22.58%	12.90%
GLN	132	59	25	0	7	8	61	0	45.21%	8.56%	20.21%	23.63%	2.40%
GLU	182	65	66	0	5	1	76	0	46.08%	16.71%	16.46%	19.49%	1.27%
HIS	2	4	0	0	7	1	2	0	12.50%	0.00%	25.00%	18.75%	43.75%
ILE	176	59	30	5	69	1	83	1	41.51%	7.08%	13.92%	20.05%	17.45%
LEU	1576	614	548	38	509	134	990	32	35.49%	12.34%	13.83%	26.03%	12.32%
LYS	76	27	17	0	0	0	20	0	54.29%	12.14%	19.29%	14.29%	0.00%
MET	63	23	13	18	2	0	27	0	43.15%	8.90%	15.75%	18.49%	13.70%
PHE	10	2	22	0	13	4	42	0	10.75%	23.66%	2.15%	49.46%	13.98%
PRO	122	38	30	0	1	38	38	0	45.69%	11.24%	14.23%	28.46%	0.37%
SER	26	2	7	0	1	0	41	0	33.77%	9.09%	2.60%	53.25%	1.30%
THR	31	7	1	0	1	0	1	0	75.61%	2.44%	17.07%	2.44%	2.44%
TRP	8	3	2	0	0	0	13	0	30.77%	7.69%	11.54%	50.00%	0.00%
TYR	26	17	13	4	45	0	12	0	22.22%	11.11%	14.53%	10.26%	41.88%
VAL	74	39	25	0	12	0	7	0	47.13%	15.92%	24.84%	4.46%	7.64%
Total	2645	1011	812	67	692	218	1432	33	38.28%	11.75%	14.63%	24.36%	10.98%

## Supplementary Materials

The following are available online, Table S1: The secondary structure of the outlier residue in X-ray-1.5, Table S2: The secondary structure of the outlier residue in EM-0-4-2016, Table S3: The secondary structure of the outlier residue in EM-0-4-2018, Table S4: The secondary structure of the outlier residue in EM-4-6-2016, Table S5: The secondary structure of the outlier residue in EM-4-6-2018, Table S6: The secondary structure of the outlier residue in EM-4-6-2019.
