# Supplementary material for: Outlier Profiles of Atomic Structures Derived from X-ray Crystallography and from Cryo-Electron Microscopy
Source: Molecules. 2020 Mar 28;25(7):1540. doi: 10.3390/molecules25071540 (PMC7181022; doi:10.3390/molecules25071540)
Supplement: Supplementary file 1 [file molecules-25-01540-s001.pdf]

Table S1. The secondary structure of the outlier residue in X-ray-1.5

	Loop			Sheet		Helix			-	T	S	Helix	Sheet
	-	S	T	B	E	G	H	I					
ARG	14	15	5	0	2	2	0	0	36.84%	13.16%	39.47%	5.26%	5.26%
ASN	18	11	25	1	3	5	3	0	27.27%	37.88%	16.67%	12.12%	6.06%
ASP	37	28	33	0	14	11	26	3	24.34%	21.71%	18.42%	26.32%	9.21%
CYS	17	1	8	0	11	1	4	0	40.48%	19.05%	2.38%	11.90%	26.19%
GLN	25	9	14	0	9	6	10	1	33.78%	18.92%	12.16%	22.97%	12.16%
GLU	60	32	37	0	11	12	18	0	35.29%	21.76%	18.82%	17.65%	6.47%
HIS	7	8	7	1	0	4	5	0	21.88%	21.88%	25.00%	28.13%	3.13%
ILE	30	7	18	4	17	2	11	0	33.71%	20.22%	7.87%	14.61%	23.60%
LEU	61	24	23	0	46	7	34	1	31.12%	11.73%	12.24%	21.43%	23.47%
LYS	25	15	10	0	8	2	11	0	35.21%	14.08%	21.13%	18.31%	11.27%
MET	9	4	5	0	3	2	7	0	30.00%	16.67%	13.33%	30.00%	10.00%
PHE	7	7	2	0	5	0	2	0	30.43%	8.70%	30.43%	8.70%	21.74%
PRO	49	22	23	0	6	5	8	0	43.36%	20.35%	19.47%	11.50%	5.31%
SER	41	27	21	0	20	1	19	0	31.78%	16.28%	20.93%	15.50%	15.50%
THR	31	15	10	0	18	3	4	1	37.80%	12.20%	18.29%	9.76%	21.95%
TRP	0	1	0	0	2	1	2	0	0.00%	0.00%	16.67%	50.00%	33.33%
TYR	1	11	4	0	6	1	7	0	3.33%	13.33%	36.67%	26.67%	20.00%
VAL	22	11	12	0	20	3	7	0	29.33%	16.00%	14.67%	13.33%	26.67%
Total	454	248	257	6	201	68	178	6	32.02%	18.12%	17.49%	17.77%	14.60%

Table S2. The secondary structure of the outlier residue in EM-0-4-2016

	Loop			Sheet		Helix			-	T	S	Helix	Sheet
	-	S	T	B	E	G	H	I					
ARG	29	22	14	0	1	15	7	0	32.95%	15.91%	25.00%	25.00%	1.14%
ASN	13	7	2	0	0	0	1	0	56.52%	8.70%	30.43%	4.35%	0.00%
ASP	12	3	2	0	1	2	15	0	34.29%	5.71%	8.57%	48.57%	2.86%
CYS	10	4	6	0	9	0	2	1	31.25%	18.75%	12.50%	9.38%	28.13%
GLN	78	24	21	5	18	18	51	0	36.28%	9.77%	11.16%	32.09%	10.70%
GLU	93	111	53	0	3	2	89	1	26.42%	15.06%	31.53%	26.14%	0.85%
HIS	1	3	0	0	0	1	3	0	12.50%	0.00%	37.50%	50.00%	0.00%
ILE	337	103	48	6	57	14	126	5	48.42%	6.90%	14.80%	20.83%	9.05%
LEU	291	145	149	12	108	30	248	17	29.10%	14.90%	14.50%	29.50%	12.00%
LYS	69	38	15	0	11	2	7	0	48.59%	10.56%	26.76%	6.34%	7.75%
MET	56	43	20	9	5	3	32	0	33.33%	11.90%	25.60%	20.83%	8.33%
PHE	20	9	2	0	8	0	3	0	47.62%	4.76%	21.43%	7.14%	19.05%
PRO	85	38	14	0	2	11	26	0	48.30%	7.95%	21.59%	21.02%	1.14%
SER	15	3	1	0	0	0	2	0	71.43%	4.76%	14.29%	9.52%	0.00%
THR	72	12	7	0	33	1	10	0	53.33%	5.19%	8.89%	8.15%	24.44%
TRP	27	1	2	0	3	4	16	0	50.94%	3.77%	1.89%	37.74%	5.66%

TYR	6	9	12	0	15	3	15	0	10.00%	20.00%	15.00%	30.00%	25.00%
VAL	191	50	22	2	29	2	41	6	55.69%	6.41%	14.58%	14.29%	9.04%
Total	1405	625	390	34	303	108	694	30	39.15%	10.87%	17.41%	23.18%	9.39%

Table S3. The secondary structure of the outlier residue in EM-0-4-2018

	Loop			Sheet		Helix			-	T	S	Helix	Sheet
	-	S	T	B	E	G	H	I					
ARG	12	9	2	0	0	0	5	0	42.86%	7.14%	32.14%	17.86%	0.00%
ASN	3	1	0	0	0	0	1	0	60.00%	0.00%	20.00%	20.00%	0.00%
ASP	5	7	4	0	2	0	5	0	21.74%	17.39%	30.43%	21.74%	8.70%
CYS	7	1	1	0	1	0	1	0	63.64%	9.09%	9.09%	9.09%	9.09%
GLN	21	12	4	0	2	1	10	0	42.00%	8.00%	24.00%	22.00%	4.00%
GLU	50	22	19	0	17	0	17	1	39.68%	15.08%	17.46%	14.29%	13.49%
HIS	0	0	0	0	0	0	1	0	0.00%	0.00%	0.00%	100.00%	0.00%
ILE	73	26	11	1	7	2	24	0	50.69%	7.64%	18.06%	18.06%	5.56%
LEU	280	172	61	4	103	18	162	0	35.00%	7.63%	21.50%	22.50%	13.38%
LYS	16	6	9	0	1	1	5	0	42.11%	23.68%	15.79%	15.79%	2.63%
MET	7	4	3	0	0	1	7	0	31.82%	13.64%	18.18%	36.36%	0.00%
PHE	4	1	1	0	2	0	4	1	30.77%	7.69%	7.69%	38.46%	15.38%
PRO	19	35	8	0	4	1	15	0	23.17%	9.76%	42.68%	19.51%	4.88%
SER	1	0	1	0	0	0	0	0	50.00%	50.00%	0.00%	0.00%	0.00%
THR	3	2	4	0	0	0	0	0	33.33%	44.44%	22.22%	0.00%	0.00%
TRP	4	1	0	0	0	0	2	0	57.14%	0.00%	14.29%	28.57%	0.00%
TYR	5	14	2	0	5	0	8	0	14.71%	5.88%	41.18%	23.53%	14.71%
VAL	49	12	8	0	2	0	8	0	62.03%	10.13%	15.19%	10.13%	2.53%
Total	559	325	138	5	146	24	275	2	37.92%	9.36%	22.05%	20.42%	10.24%

Table S4. The secondary structure of the outlier residue in EM-4-6-2016

	Loop			Sheet		Helix			-	T	S	Helix	Sheet
	-	S	T	B	E	G	H	I					
ARG	42	20	39	0	2	1	26	2	31.82%	29.55%	15.15%	21.97%	1.52%
ASN	17	18	10	0	6	1	31	0	20.48%	12.05%	21.69%	38.55%	7.23%
ASP	53	15	18	3	8	6	37	0	37.86%	12.86%	10.71%	30.71%	7.86%
CYS	33	2	4	0	20	0	3	0	53.23%	6.45%	3.23%	4.84%	32.26%
GLN	67	28	8	2	22	2	11	0	47.86%	5.71%	20.00%	9.29%	17.14%
GLU	127	101	46	1	4	1	18	1	42.47%	15.38%	33.78%	6.69%	1.67%
HIS	24	18	14	0	17	3	10	2	27.27%	15.91%	20.45%	17.05%	19.32%
ILE	241	125	33	3	24	6	58	1	49.08%	6.72%	25.46%	13.24%	5.50%
LEU	250	124	72	7	66	16	223	3	32.85%	9.46%	16.29%	31.80%	9.59%
LYS	100	42	28	0	6	6	20	0	49.50%	13.86%	20.79%	12.87%	2.97%
MET	39	33	9	2	5	0	9	0	40.21%	9.28%	34.02%	9.28%	7.22%
PHE	72	49	16	0	59	3	52	0	28.69%	6.37%	19.52%	21.91%	23.51%
PRO	110	95	30	1	10	16	22	0	38.73%	10.56%	33.45%	13.38%	3.87%
SER	30	9	7	0	4	3	9	0	48.39%	11.29%	14.52%	19.35%	6.45%
THR	61	17	9	0	32	9	34	0	37.65%	5.56%	10.49%	26.54%	19.75%

TRP	11	0	14	0	17	4	28	0	14.86%	18.92%	0.00%	43.24%	22.97%
TYR	46	39	19	8	26	2	28	2	27.06%	11.18%	22.94%	18.82%	20.00%
VAL	122	46	45	4	56	18	30	0	38.01%	14.02%	14.33%	14.95%	18.69%
Total	1445	781	421	31	384	97	649	11	37.84%	11.02%	20.45%	19.82%	10.87%

Table S5. The secondary structure of the outlier residue in EM-4-6-2018

	Loop			Sheet		Helix			-	T	S	Helix	Sheet
	-	S	T	B	E	G	H	I					
ARG	9	15	9	0	0	0	6	0	23.08%	23.08%	38.46%	15.38%	0.00%
ASN	13	4	3	0	6	0	14	0	32.50%	7.50%	10.00%	35.00%	15.00%
ASP	25	6	11	0	16	1	20	0	31.65%	13.92%	7.59%	26.58%	20.25%
CYS	4	1	0	0	5	0	2	0	33.33%	0.00%	8.33%	16.67%	41.67%
GLN	25	8	13	0	2	1	12	0	40.98%	21.31%	13.11%	21.31%	3.28%
GLU	45	31	39	1	18	0	46	1	24.86%	21.55%	17.13%	25.97%	10.50%
HIS	3	4	1	0	8	0	9	0	12.00%	4.00%	16.00%	36.00%	32.00%
ILE	65	39	5	0	9	6	16	0	46.43%	3.57%	27.86%	15.71%	6.43%
LEU	177	85	42	3	38	2	148	2	35.61%	8.45%	17.10%	30.58%	8.25%
LYS	21	6	3	0	0	0	5	0	60.00%	8.57%	17.14%	14.29%	0.00%
MET	30	11	9	0	0	0	10	0	50.00%	15.00%	18.33%	16.67%	0.00%
PHE	20	10	8	1	22	0	21	0	24.39%	9.76%	12.20%	25.61%	28.05%
PRO	38	14	3	0	1	0	22	0	48.72%	3.85%	17.95%	28.21%	1.28%
SER	5	7	1	6	3	0	6	0	17.86%	3.57%	25.00%	21.43%	32.14%
THR	7	6	6	2	9	5	9	0	15.91%	13.64%	13.64%	31.82%	25.00%
TRP	10	0	7	0	2	0	7	0	38.46%	26.92%	0.00%	26.92%	7.69%
TYR	13	4	7	0	24	0	6	1	23.64%	12.73%	7.27%	12.73%	43.64%
VAL	32	18	2	1	44	1	29	0	25.20%	1.57%	14.17%	23.62%	35.43%
Total	542	269	169	14	207	16	388	4	33.69%	10.50%	16.72%	25.36%	13.74%

Table S6. The secondary structure of the outlier residue in EM-4-6-2019

	Loop			Sheet		Helix			-	T	S	Helix	Sheet
	-	S	T	B	E	G	H	I					
ARG	27	3	10	0	0	4	6	0	54.00%	20.00%	6.00%	20.00%	0.00%
ASN	0	11	1	0	0	1	15	0	0.00%	3.57%	39.29%	57.14%	0.00%
ASP	6	23	2	0	3	0	3	0	16.22%	5.41%	62.16%	8.11%	8.11%
CYS	5	2	1	0	3	0	2	0	38.46%	7.69%	15.38%	15.38%	23.08%
GLN	48	10	12	4	1	3	10	0	54.55%	13.64%	11.36%	14.77%	5.68%
GLU	86	60	48	2	0	1	26	0	38.57%	21.52%	26.91%	12.11%	0.90%
HIS	0	0	0	0	5	0	0	0	0.00%	0.00%	0.00%	0.00%	100.00%
ILE	120	35	23	9	29	2	32	4	47.24%	9.06%	13.78%	14.96%	14.96%

LEU	534	251	210	11	180	26	500	11	30.99%	12.19%	14.57%	31.17%	11.09%
LYS	36	5	8	0	0	0	19	0	52.94%	11.76%	7.35%	27.94%	0.00%
MET	31	4	2	0	1	1	17	4	51.67%	3.33%	6.67%	36.67%	1.67%
PHE	2	28	4	0	6	0	12	0	3.85%	7.69%	53.85%	23.08%	11.54%
PRO	29	4	7	0	2	0	10	0	55.77%	13.46%	7.69%	19.23%	3.85%
SER	2	0	0	0	0	0	0	0	100.00%	0.00%	0.00%	0.00%	0.00%
THR	4	1	1	0	3	5	0	0	28.57%	7.14%	7.14%	35.71%	21.43%
TRP	5	0	3	0	0	0	10	0	27.78%	16.67%	0.00%	55.56%	0.00%
TYR	14	6	3	1	23	1	10	0	24.14%	5.17%	10.34%	18.97%	41.38%
VAL	50	16	16	0	4	0	55	0	35.46%	11.35%	11.35%	39.01%	2.84%
Total	999	459	351	27	260	44	727	19	34.62%	12.16%	15.90%	27.37%	9.94%
